# Effects of Bilateral Robotic Arm Training in Stroke Patients: A Systematic Review and Meta-Analysis

**DOI:** 10.3390/medsci14020293

**Published:** 2026-06-05

**Authors:** Sasithorn Khawprapa, Nuttaset Manimmanakorn, Yohei Otaka, Jittima Saengsuwan

**Affiliations:** 1 Graduate School, Faculty of Medicine, Khon Kaen University, Khon Kaen 40002, Thailand; 2 Department of Rehabilitation Medicine, Graduate School of Medicine, Fujita Health University, Toyoake 470-1192, Aichi, Japan; 3 Department of Rehabilitation Medicine, Faculty of Medicine, Khon Kaen University, Khon Kaen 40002, Thailand; 4 Department of Rehabilitation Medicine, School of Medicine, Fujita Health University, Toyoake 470-1192, Aichi, Japan

**Keywords:** stroke rehabilitation, bilateral robotic training, upper limb, meta-analysis

## Abstract

**Objectives**: Bilateral robotic arm training (BRT) may enhance poststroke motor recovery by reducing interhemispheric inhibition and promoting bilateral motor network engagement. However, previous reviews have often pooled bilateral and unilateral robotic approaches, potentially masking differential effects. This systematic review and meta-analysis compared the effects of BRT with those of unilateral robotic training (URT) and conventional rehabilitation on upper-limb motor function after stroke. **Methods**: Randomized controlled trials were identified through systematic searches of major electronic databases and trial registries in accordance with PRISMA guidelines. The risk of bias was assessed via the Cochrane Risk of Bias 2 tool. Random effects meta-analyses were performed using standardized mean differences (SMDs). Predefined subgroup and sensitivity analyses were used to examine the influence of participant characteristics, training dose, intervention duration, and robotic device type. **Results**: Fourteen randomized controlled trials involving 440 participants were included. Overall, compared with control interventions, BRT did not significantly improve upper-limb motor function, as measured using the Fugl–Meyer Assessment for Upper Extremity (SMD = 0.18, 95% CI −0.01–0.36). Significant effects were observed in participants younger than 60 years, with training doses > 15 h, intervention durations > 4 weeks, and use of Bi-Manu-Track systems. **Conclusions**: BRT did not demonstrate a significant overall advantage over URT or conventional rehabilitation. However, subgroup analyses suggest that treatment effects may vary according to patient characteristics, training dose, duration of the intervention, and device type.

## 1. Introduction

The Global Burden of Disease study revealed that despite reductions in age-standardized incidence and mortality, the absolute burden of stroke continues to increase worldwide [1,2]. More than 70% of stroke survivors experience upper-limb weakness, which affects their ability to perform activities of daily living and engage in social interactions, resulting in psychological impacts [3,4,5,6,7]. Rehabilitation of upper-limb weakness to improve strength and functional capacity is therefore essential. Among the available rehabilitation strategies, robotic-assisted therapy has emerged as an important and influential approach [8,9].

Robotic assistance in upper-limb rehabilitation contributes to increased muscle strength; enhanced sensory perception, coordination, and flexibility; and reduced spasticity. It also improves joint mobility, shortens the rehabilitation period, and enhances exercise consistency for patients, thereby improving their overall quality of life [10].

Currently, there are two main robotic-assisted approaches: unilateral robotic training (URT) and bilateral robotic training (BRT). These two robotic systems have the potential to increase upper-limb strength and functional capacity. However, compared with unilateral robotic-assisted training, bilateral robotic-assisted therapy has been shown to yield superior outcomes in the context of muscle strength enhancement and the facilitation of activities of daily living [11,12].

Neurophysiological investigations of stroke patients have revealed that the primary motor cortex in the unaffected hemisphere functions as an inhibitory signaling mechanism toward the primary motor cortex in the affected hemisphere. This abnormal inhibition can negatively impact the recovery of muscle strength in stroke patients [13]. A previous study demonstrated that engaging in bilateral arm movements resulted in facilitation in both hemispheres. Furthermore, they reported that after a short training period, bilateral training reduced intracortical inhibition and increased intracortical facilitation in both hemispheres. In contrast, unilateral training primarily increased intracortical facilitation and decreased intracortical inhibition, but these effects were limited to the contralateral hemisphere [14]. This may explain the reported functional benefits of BRT. Nevertheless, findings from randomized trials remain inconsistent: whereas some studies have shown significant improvements in arm function with BRT, others have reported no clear difference compared with URT or conventional therapy [15,16,17].

Previous systematic reviews of robotic-assisted therapy for poststroke upper-limb rehabilitation have reported inconsistent findings [18,19]. However, these studies often analyzed bilateral and unilateral robotic training together as a single intervention category compared with conventional rehabilitation, despite potential differences in their mechanisms and functional effects [14]. Moreover, recent randomized controlled trials have provided new comparative data [20,21], highlighting the need for focused systematic reviews and meta-analyses to clarify their relative effectiveness.

Therefore, the objective of this systematic review and meta-analysis was to evaluate the comparative effectiveness of BRT versus unilateral robotic training and/or conventional rehabilitation on upper-extremity motor function after stroke.

## 2. Materials and Methods

### 2.1. Protocol Registration

This systematic review was conducted in accordance with the PRISMA guidelines [22] and was registered in PROSPERO (CRD42024547178). The review protocol is publicly available through the PROSPERO database. No major amendments were made to the original protocol after registration.

### 2.2. Search Strategy

We searched Cochrane, MEDLINE, Scopus, PEDro, Google Scholar, Embase and clinical trial registries; the final search was completed on 24 May 2025. We conducted a comprehensive search using keywords related to stroke, interventions, and upper-limb outcomes. The stroke-related terms included stroke, hemiparesis, upper-extremity impairment, upper-extremity paresis, upper-limb motor deficits, and cerebrovascular accidents. Intervention-related terms included robot-assisted upper-limb therapy, robot rehabilitation, bilateral robotic, bilateral priming, bilateral hybrid, hybrid rehabilitation, robot-assisted upper limb, robot-assisted movement training, robot-assisted bilateral arm training, and robotic device training. The outcome-related terms included Fugl–Meyer Assessment, upper-extremity motor function, upper-limb function, Fugl–Meyer Assessment Scale, upper-extremity performance, motor activity, and recovery of function. All the search terms were adapted for each database, and the full search strategies are provided in Supplement S1.

### 2.3. Eligibility Criteria

The eligibility criteria for this review, defined according to the PICOS (Population, Intervention, Comparison, Outcomes, and Study design) framework, were as follows:

Population: Adult patients (aged ≥18 years) with stroke and upper-limb motor impairment.

Intervention: Bilateral robotic arm training.

Comparison: Unilateral robotic arm training or conventional rehabilitation. For studies with multiple control groups, data were combined into a single comparator group. Although unilateral robotic training and conventional rehabilitation differ conceptually in terms of technology and task specificity, they were pooled as non-bilateral control conditions to enable a pragmatic comparison with BRT. To address potential clinical heterogeneity, subgroup analyses stratified by comparator type were conducted to assess the consistency of treatment effects across different control conditions.

Outcomes: The primary outcome was upper-extremity motor function, which was assessed via the Fugl–Meyer Assessment for the Upper Extremity (FMA-UE). The secondary outcomes included activities of daily living, which were measured via the Functional Independence Measure (FIM) and the Motor Activity Log (MAL).

Study design: Only randomized controlled trials (RCTs) were included.

### 2.4. Study Selection and Data Collection

Two independent reviewers initially screened the titles and abstracts. The full-text articles were then assessed according to the inclusion criteria for study design, participants, interventions, and outcomes. Any disagreements were resolved through discussion, and if a consensus was not reached, a third reviewer was consulted.

A standardized data extraction form was developed, and two reviewers independently extracted all relevant information. The extracted data included study characteristics (authors, publication year, country, and sample size) and sample characteristics (sex, age, type of intervention and comparator, intervention frequency, duration, intensity, time since stroke onset, and inclusion/exclusion criteria). The outcome measures collected were the Fugl–Meyer Assessment–Upper Extremity section, the Motor Activity Log (MAL), and the Functional Independence Measure (FIM). We also recorded the timing of the outcome assessments (baseline, postintervention, and follow-up), as well as any adverse events and reasons for participant dropout.

### 2.5. Risk of Bias and Quality Assessment

The risk of bias assessment for the included studies was conducted independently by two review authors in accordance with the criteria specified in the Cochrane Handbook for Systematic Reviews of Interventions. The tool assesses domain-specific quality across five areas: bias arising from the randomization process, bias due to deviations from intended interventions, bias due to missing outcome data, bias in the measurement of outcomes, and bias in the selection of reported results. Each study was rated as having a “low risk of bias,” “high risk of bias,” or “some concerns.”

Publication bias was assessed through visual inspection of funnel plots for the primary outcome. To provide a quantitative assessment of funnel plot asymmetry, Egger’s linear regression test was performed. When potential publication bias was suspected, the Duval and Tweedie trim-and-fill method was applied to estimate the potential impact of missing studies on the pooled effect size.

The certainty of evidence for the primary outcome was assessed via the Grading of Recommendations Assessment, Development and Evaluation (GRADE) framework. Each outcome was classified as having high, moderate, low, or very low certainty on the basis of five domains: risk of bias, inconsistency, indirectness, imprecision, and publication bias. Decisions to downgrade the certainty of evidence were made according to the severity of limitations identified within these domains.

### 2.6. Data Synthesis and Statistical Analysis

Meta-analyses were performed using R software (version 4.5.2) with the meta package. Pre- to postintervention mean changes and corresponding standard deviations were used to calculate standardized mean differences (SMDs) between the BRT and control groups. Although all the studies assessed upper-limb motor impairment via the Fugl–Meyer assessment, SMDs were calculated to account for substantial variability in baseline severity and outcome dispersion across the studies.

When standard deviations were not reported but standard errors were available, missing SDs were derived using the formula SD = SE × √n. A random-effects model was applied a priori to account for expected clinical and methodological heterogeneity across the included studies, including differences in participant characteristics, types of BRT devices, and total intervention dose. Pooled effect estimates are presented with 95% confidence intervals.

Statistical heterogeneity was assessed via Tau^2^, I^2^, and the Chi^2^ test. Substantial heterogeneity was defined as an I^2^ value greater than 50% or a statistically significant Chi^2^ test (*p* < 0.10), whereas Tau^2^ was reported as a measure of between-study variance [23].

Subgroup analyses for the primary outcome were conducted using study-level characteristics on the basis of summary values reported for each trial where sufficient data were available. Stratification was performed according to age (<60 vs. ≥60 years) [24,25], baseline stroke severity (severe: FMA-UE < 30; mild/moderate: FMA-UE ≥ 30) [26], and type of BRT device. Furthermore, the total training dose was dichotomized at a 15 h threshold [27], while the duration of the intervention was categorized as <4 weeks or ≥4 weeks based on the previous literature [28].

Sensitivity analyses were conducted to examine the robustness of the pooled effect estimates. In addition, sensitivity analyses were performed by excluding studies assessed as having a high risk of bias to evaluate the stability of the findings.

## 3. Results

### 3.1. Database Screening

**Figure 1:**
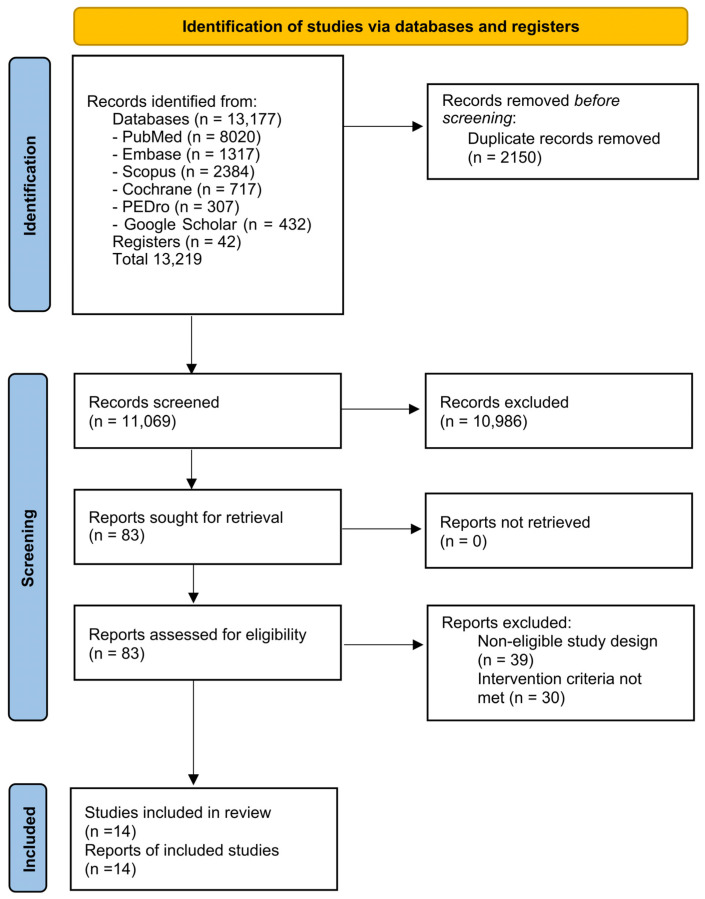
Study selection results.

The search identified 13,219 records. After the removal of duplicates, 11,069 records were screened, and 83 full-text reports were assessed for eligibility. Sixty-nine reports were excluded, and 14 randomized controlled trials were included in the qualitative and quantitative synthesis (Figure 1). The inter-rater agreement during the study selection phase yielded a Cohen’s kappa coefficient of 0.79.

### 3.2. Study Characteristics

Fourteen randomized controlled trials were included, with sample sizes ranging from 12 to 70 and mean participant ages between 51 and 70 years. The participants were in the subacute or chronic phases of stroke recovery and exhibited moderate-to-severe upper-limb impairment (baseline FMA-UE score: 7.9–44.9). All trials investigated BRT compared with control interventions. The training dosage ranged from 12 to 45 h over 3–8 weeks, with the intensity generally matching between the groups. Upper-limb motor impairment was primarily assessed via the FMA-UE, with activities of daily living evaluated as secondary outcomes. The detailed study characteristics are summarized in Table 1. Regarding safety and feasibility, most studies reported no serious adverse events, with only a few instances of mild fatigue or transient musculoskeletal pain. Post-intervention dropout rates ranged from 0% to 11.4%, primarily due to reasons unrelated to the study protocols, such as medical complications or refusal to undergo evaluation (Supplementary Table S1).

### 3.3. Characteristics of the Interventions

The characteristics of the interventions are summarized in Table 2.

#### 3.3.1. Mirror Image Movement Enabler (MIME)

The mirror image movement enabler (MIME) is an end-effector–based robotic system that enables three-dimensional upper-limb reaching. Training focuses on proximal arm control through restricted wrist and hand motion and includes passive, active-assisted, active-constrained, and bimanual mirror modes. Real-time mirroring of the nonparetic limb supports bilateral practice, with safety ensured through continuous force monitoring and integrated safeguards [17,29,31].

#### 3.3.2. Bi-Manu-Track System

The Bi-Manu-Track system is a bilateral robotic device designed to facilitate repetitive, mirror-symmetric upper-limb movements in individuals with hemiparesis. It enables bilateral training of forearm pronation–supination and wrist flexion–extension, with the forearms stabilized to ensure consistent joint alignment. The device provides passive–passive, active–passive, and active–active training modes, allowing graded progression from passive to active bilateral practice. Safety is ensured through built-in mechanical limits and emergency stop mechanisms [16,39].

#### 3.3.3. Bilateral Upper-Limb Rehabilitation Robot (ESTUN)

The ESTUN bilateral upper-limb rehabilitation robot is a three-dimensional end-effector system designed to support shoulder and elbow movements during upper-limb training. In the included studies, both unilateral and BRT protocols were applied, typically in combination with task-oriented functional exercises. The detailed intervention parameters are provided in the Supplementary Material [20,40].

#### 3.3.4. Arm Light Exoskeleton Rehab Station (ALEx RS)

The ALEx RS is a wearable upper-limb exoskeleton system that can be used in unilateral or bilateral training configurations. It supports three-dimensional arm movements with automated assistance to facilitate task completion. Training protocols combine proximal joint mobilization with goal-oriented virtual reality-based exergames targeting reaching, object manipulation, and visuomotor coordination. In bilateral mode, movements of the nonparetic limb are mirrored in real time to the affected limb to enable coordinated bimanual practice [21,41].

### 3.4. Risk of Bias Assessment

**Figure 2:**
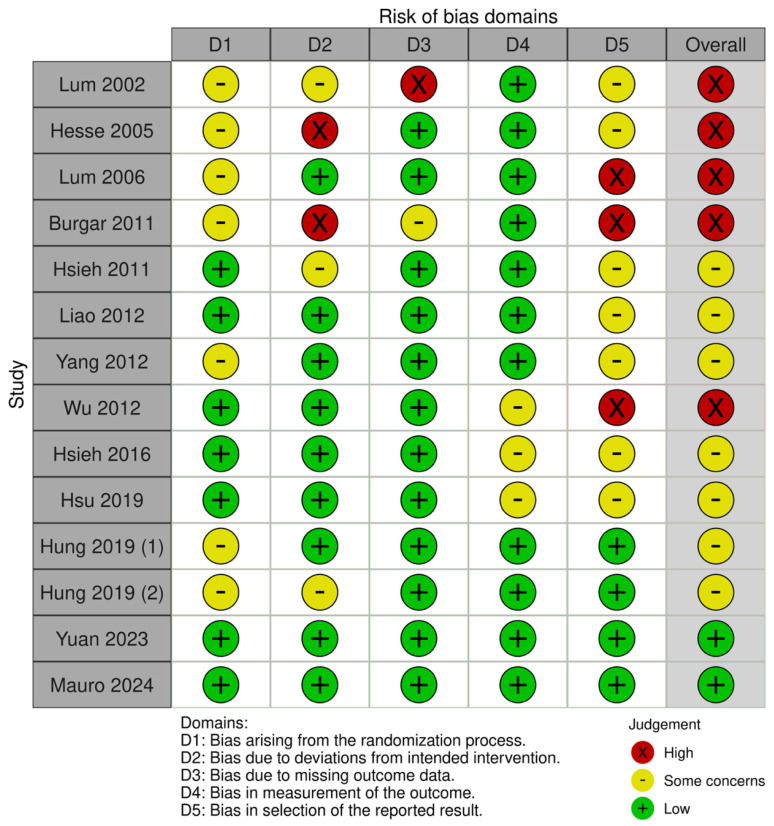
Risk of bias assessment of the included randomized controlled trials via the RoB 2 tool. Studies included: Lum 2002 [29], Hesse 2005 [30], Lum 2006 [31], Burgar 2011 [17], Hsieh 2011 [32], Liao 2012 [33], Yang 2012 [35], Wu 2012 [34], Hsieh 2016 [36], Hsu 2019 [37], Hung 2019 (1) [38], Hung 2019 (2) [15], Yuan 2023 [20], and Mauro 2024 [21].

**Figure 3:**
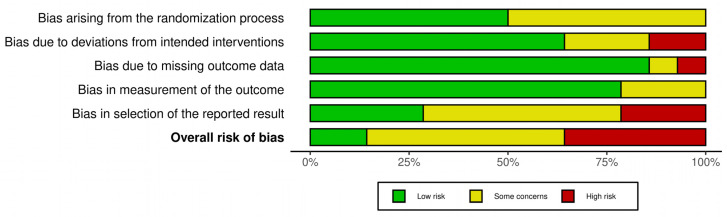
Assessment of risk of bias presented as percentages across all included studies.

The risk of bias assessment revealed variability in methodological quality across studies. Although most trials demonstrated a low risk of bias in randomization and missing outcome data, concerns were frequently identified in deviations from intended interventions and outcome measurement, primarily owing to challenges in blinding. Earlier studies were more often rated as high risk or with some concerns overall, whereas more recent trials generally demonstrated a low risk of bias across domains (Figure 2). Overall, most studies had a low risk of bias for missing outcome data and outcome measurement, whereas greater concerns were observed in deviations from intended interventions and selection of the reported results, with a higher proportion of trials rated as having some concerns or high risk (Figure 3).

### 3.5. Meta-Analysis Findings

#### Comparative Effects of Interventions

**Figure 4:**
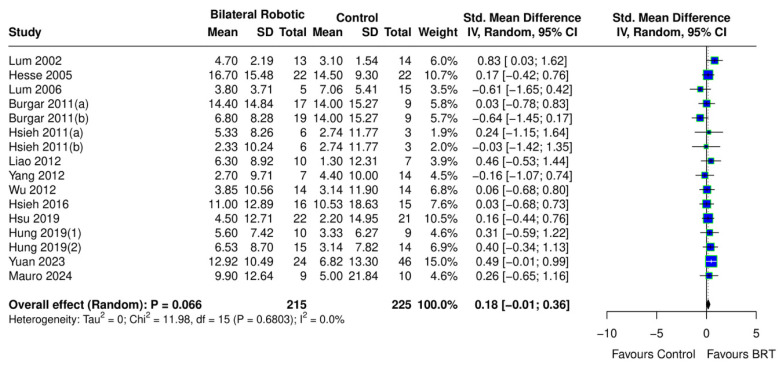
Forest plot comparing the effects of bilateral robotic training and control interventions on upper-limb motor recovery measured by the FMA-UE. Studies included: Lum 2002 [29], Hesse 2005 [30], Lum 2006 [31], Burgar 2011 [17], Hsieh 2011 [32], Liao 2012 [33], Yang 2012 [35], Wu 2012 [34], Hsieh 2016 [36], Hsu 2019 [37], Hung 2019 (1) [38], Hung 2019 (2) [15], Yuan 2023 [20], and Mauro 2024 [21].

**Figure 5:**
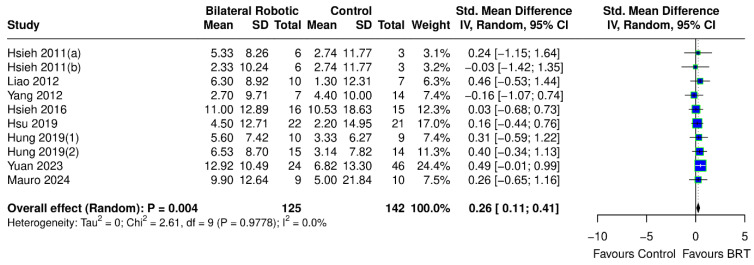
Forest plot comparing the effects of bilateral robotic training and control interventions on upper-limb motor recovery, excluding studies at high risk of bias. Studies included: Hsieh 2011 [32], Liao 2012 [33], Yang 2012 [35], Hsieh 2016 [36], Hsu 2019 [37], Hung 2019 (1) [38], Hung 2019 (2) [15], Yuan 2023 [20], and Mauro 2024 [21].

**Figure 6:**
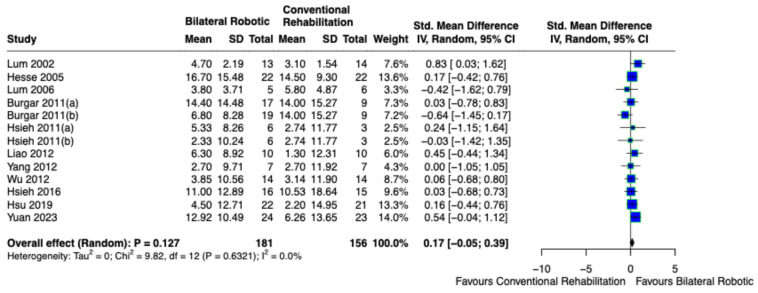
Forest plot comparing the effects of bilateral robotic training and conventional rehabilitation on upper-limb motor recovery as measured by the FMA-UE. Studies included: Lum 2002 [29], Hesse 2005 [30], Lum 2006 [31], Burgar 2011 [17], Hsieh 2011 [32], Liao 2012 [33], Yang 2012 [35], Wu 2012 [34], Hsieh 2016 [36], Hsu 2019 [37] and Yuan 2023 [20].

**Figure 7:**
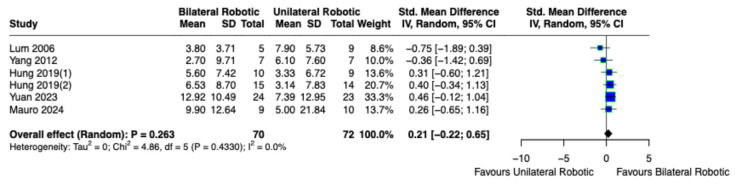
Forest plot comparing the effects of bilateral robotic training and unilateral robotic training on upper-limb motor recovery as measured by the FMA-UE. Studies included: Lum 2002 [29], Yang 2012 [35], Hung 2019 (1) [38], Hung 2019 (2) [15], Yuan 2023 [20], and Mauro 2024 [21].

The pooled meta-analysis demonstrated no statistically significant difference between BRT and control interventions for upper-limb motor function recovery measured by the FMA-UE (SMD = 0.18, 95% CI [−0.01, 0.36], *p* = 0.066) (Figure 4). When studies assessed as high risk of bias were excluded, sensitivity analysis revealed a significant positive effect of BRT (SMD = 0.26, 95% CI [0.11, 0.41], *p* = 0.004) (Figure 5). Subgroup analyses stratified by comparator type (bilateral robotic training vs. unilateral robotic training and conventional rehabilitation) showed no statistically significant differences between groups. Corresponding forest plots are presented in Figure 6 and Figure 7.

**Figure 8:**
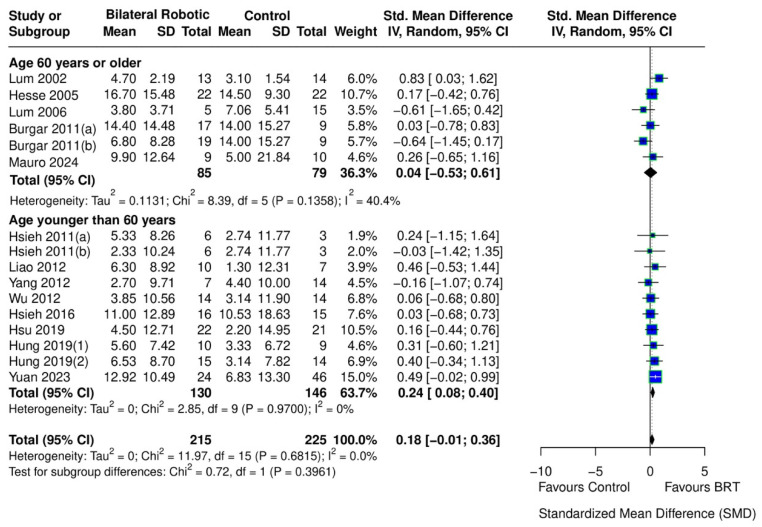
Forest plot comparing the effects of bilateral robotic training and control interventions on upper-limb motor recovery, stratified by age. Studies included: Lum 2002 [29], Hesse 2005 [30], Lum 2006 [31], Burgar 2011 [17], Hsieh 2011 [32], Liao 2012 [33], Yang 2012 [35], Wu 2012 [34], Hsieh 2016 [36], Hsu 2019 [37], Hung 2019 (1) [38], Hung 2019 (2) [15], Yuan 2023 [20], and Mauro 2024 [21].

**Figure 9:**
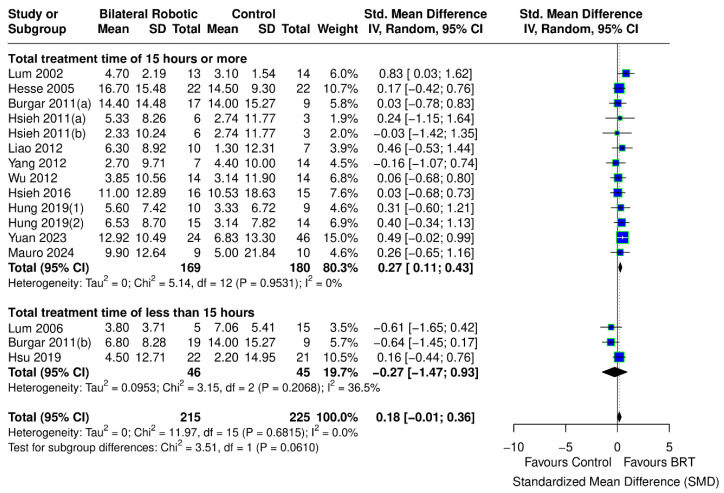
Forest plot comparing the effects of bilateral robotic training and control intervention on upper-limb motor recovery, stratified by total training dose (hours). Studies included: Lum 2002 [29], Hesse 2005 [30], Lum 2006 [31], Burgar 2011 [17], Hsieh 2011 [32], Liao 2012 [33], Yang 2012 [35], Wu 2012 [34], Hsieh 2016 [36], Hsu 2019 [37], Hung 2019 (1) [38], Hung 2019 (2) [15], Yuan 2023 [20], and Mauro 2024 [21].

**Figure 10:**
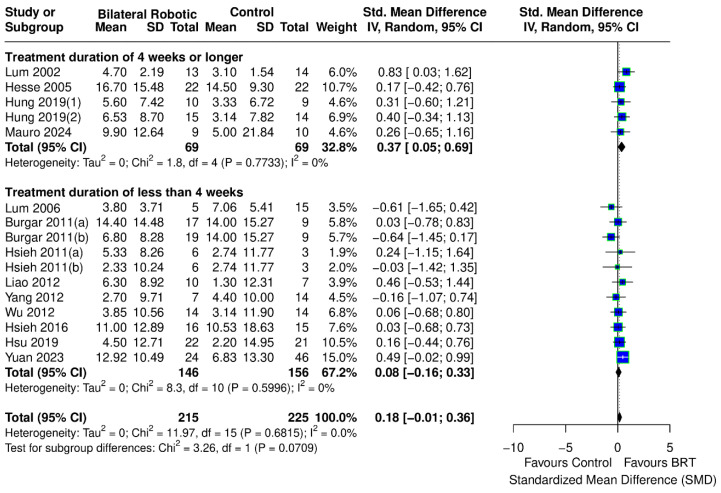
Forest plot comparing the effects of bilateral robotic training and control interventions on upper-limb motor recovery, stratified by duration of treatment (weeks). Studies included: Lum 2002 [29], Hesse 2005 [30], Lum 2006 [31], Burgar 2011 [17], Hsieh 2011 [32], Liao 2012 [33], Yang 2012 [35], Wu 2012 [34], Hsieh 2016 [36], Hsu 2019 [37], Hung 2019 (1) [38], Hung 2019 (2) [15], Yuan 2023 [20], and Mauro 2024 [21].

**Figure 11:**
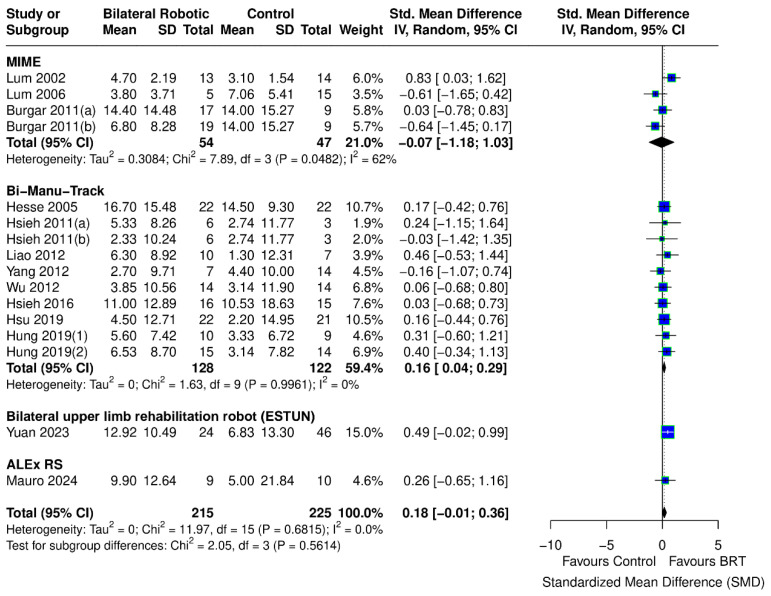
Forest plot comparing the effects of bilateral robotic training and control interventions on upper-limb motor recovery, stratified by robotic type. Studies included: Lum 2002 [29], Hesse 2005 [30], Lum 2006 [31], Burgar 2011 [17], Hsieh 2011 [32], Liao 2012 [33], Yang 2012 [35], Wu 2012 [34], Hsieh 2016 [36], Hsu 2019 [37], Hung 2019 (1) [38], Hung 2019 (2) [15], Yuan 2023 [20], and Mauro 2024 [21].

**Figure 12:**
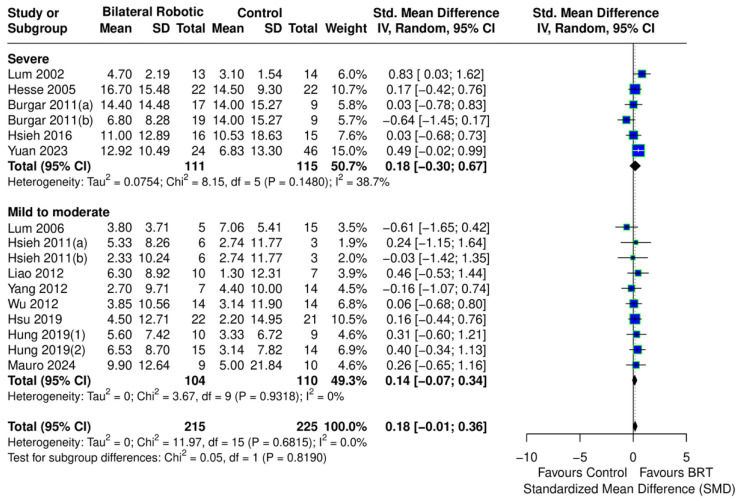
Forest plot comparing the effects of bilateral robotic training and control interventions on upper-limb motor recovery, stratified by stroke severity. Studies included: Lum 2002 [29], Hesse 2005 [30], Lum 2006 [31], Burgar 2011 [17], Hsieh 2011 [32], Liao 2012 [33], Yang 2012 [35], Wu 2012 [34], Hsieh 2016 [36], Hsu 2019 [37], Hung 2019 (1) [38], Hung 2019 (2) [15], Yuan 2023 [20], and Mauro 2024 [21].

Additional subgroup analyses were conducted to explore factors associated with the efficacy of BRT, including age, baseline impairment severity, training dose, treatment duration, and device type. Within-subgroup analyses demonstrated statistically significant pooled effects among participants younger than 60 years (SMD = 0.24; 95% CI 0.08–0.40) (Figure 8), among those in protocols delivering a total training dose exceeding 15 h (SMD = 0.27; 95% CI 0.11–0.43) (Figure 9), and among those in interventions lasting more than 4 weeks (SMD = 0.37; 95% CI 0.05–0.69) (Figure 10). A significant effect was also observed in the subgroup that received the Bi-Manu-Track system (SMD = 0.16; 95% CI 0.04–0.29) (Figure 11). No significant differences were found across baseline impairment severity levels (Figure 12), and the overall tests for subgroup differences were non-significant (*p* > 0.05). Between-study heterogeneity within the subgroups was low, which was consistent with the findings of the primary analysis.

**Figure 13:**
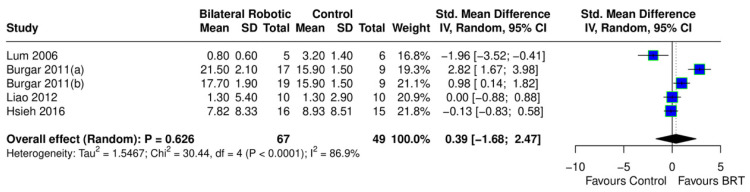
Forest plot comparing the effects of bilateral robotic training and control interventions on the functional independence measure (FIM). Studies included: Lum 2006 [31], Burgar 2011 [17], Liao 2012 [33] and Hsieh 2016 [36].

**Figure 14:**
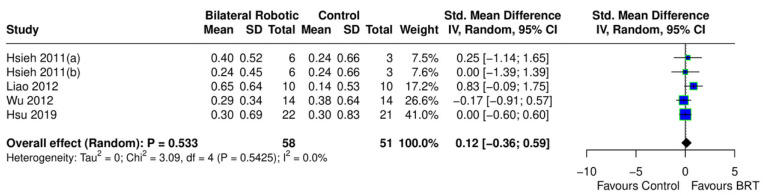
Forest plot comparing the effects of bilateral robotic training and control interventions on Motor Activity Log–Amount of Use (MAL AOU). Studies included: Hsieh 2011 [32], Liao 2012 [33], Wu 2012 [34] and Hsu 2019 [37].

**Figure 15:**
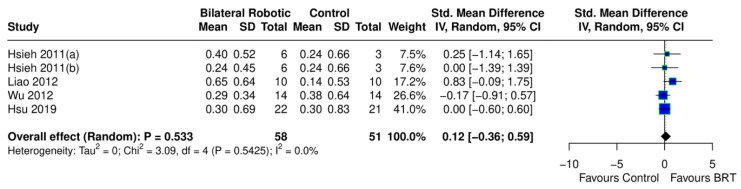
Forest plot comparing the effects of bilateral robotic training and control interventions on Motor Activity Log–Quality of Movement (MAL QOM). Studies included: Hsieh 2011 [32], Liao 2012 [33], Wu 2012 [34] and Hsu 2019 [37].

In contrast, for activities of daily living, as assessed via the functional independence measure and motor activity log, no statistically significant differences were observed between the BRT and control interventions across pooled analyses (Figure 13, Figure 14 and Figure 15).

### 3.6. Publication Bias

**Figure 16:**
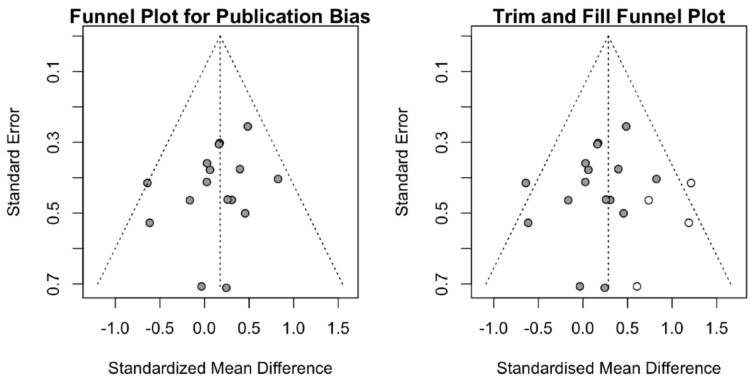
Funnel plots for the assessment of publication bias for the primary outcome.

Visual inspection of the funnel plot did not reveal substantial asymmetry. Egger’s regression test indicated no evidence of publication bias (t = −1.20, df = 14, *p* = 0.25). The results of the trim-and-fill analysis did not markedly alter the pooled effect estimate, as shown in Figure 16.

### 3.7. Effects of BRT on Upper-Limb Motor Function

#### Certainty of Evidence (GRADE)

Fourteen randomized controlled trials comprising sixteen comparisons (440 participants) evaluated upper-limb motor function via the Fugl–Meyer Assessment for the Upper Extremity (FMA-UE). Compared with the control interventions, BRT led to a small, non-significant improvement (SMD = 0.18, 95% CI [−0.01–0.36] *p* = 0.066) (Table 3). In accordance with the GRADE framework, the certainty of evidence for this outcome was rated as low.

## 4. Discussion

### 4.1. Overall Effects and Sensitivity Findings

This meta-analysis examined the effects of BRT on upper-limb motor recovery after stroke. The pooled analysis did not reveal a statistically significant overall advantage of BRT over control interventions for FMA-UE outcomes (SMD = 0.18, 95% CI −0.01–0.36). From a clinical perspective, an SMD of 0.18 represents a small effect size according to Cohen’s criteria [42], suggesting that any improvement in motor function is likely to be modest and may not reach a clinically meaningful threshold. However, the exclusion of studies at high risk of bias revealed a significant positive effect of BRT (SMD = 0.26, 95% CI 0.11–0.41), representing a small but statistically significant effect. This finding suggests that the beneficial effect of BRT may be influenced by the methodological quality of the included studies.

### 4.2. Neurophysiological Mechanisms

Neurophysiological evidence supports the observed effects under specific conditions. Neuroimaging studies using functional MRI suggest that bilateral movements can engage distributed motor networks, including activation of the contralesional motor cortex and the ipsilesional cerebellum [43]. Electrophysiological evidence from EMGs and EEGs further indicates that the modulation of muscle activation patterns and interhemispheric connectivity is relevant to motor learning and cortical reorganization [20,40]. However, although BRT may induce neural changes, these changes do not consistently translate into clear improvements in motor performance. The findings of this study suggest that the clinical benefits of BRT are variable and depend on patient characteristics, training duration, and device design rather than on bilateral training alone.

### 4.3. Influence of Robotic Device Design

Subgroup analysis stratified by robotic system revealed variability in treatment effects across devices, with the Bi-Manu-Track system being the only platform showing a significant advantage over control conditions (SMD 0.16; 95% CI 0.04–0.29). This finding is consistent with prior evidence indicating that distal-emphasizing robotic training may produce greater improvements in muscle strength and movement quality during functional activities than proximal-emphasizing robotic training [44]. Taken together, these observations suggest that rehabilitation outcomes are likely driven by device-specific mechanical and training features rather than the mere provision of robotic assistance.

The observed efficacy of the Bi-Manu-Track system may be related to its emphasis on highly repetitive, cyclic distal movements of the forearm and wrist, which may facilitate sensorimotor integration and interhemispheric interaction—mechanisms that have been associated with neuroplastic reorganization following stroke [38,45,46]. The constrained and rhythmically structured training environment of the Bi-Manu-Track system may also promote intensive, task-specific distal practice, consistent with established principles of experience-dependent plasticity and motor learning [47,48]. However, proximal-emphasizing robotic systems did not demonstrate significant advantages over control conditions. This lack of a clear effect may partly reflect the limited number of studies that have evaluated proximal-focused bilateral robotic training as well as heterogeneity in the intervention protocols across trials. Consequently, the current evidence remains insufficient to determine whether proximal-focused robotic rehabilitation provides meaningful benefits over control methods.

### 4.4. Influence of Patient Characteristics on BRT Outcomes

Subgroup analyses suggested that patient characteristics, particularly age, may modulate the therapeutic efficacy of BRT relative to control interventions. Greater improvements were observed among younger participants (<60 years; SMD 0.24; 95% CI 0.08–0.40), whereas in older participants, the pooled estimate showed a small positive effect that did not reach statistical significance. One possible explanation is that BRT relies on experience-dependent neuroplasticity and intensive motor learning [43,46,47,49]. As neural adaptability declines with advancing age—reflected by reduced neuroplastic capacity and cortical excitability [50,51,52]—younger individuals may thus exhibit greater responsiveness to repetition-based training, reflecting greater training-induced neuroplasticity [53]. With respect to baseline motor severity, no clear evidence of effect modification was observed.

### 4.5. Impact of Training Dose on BRT Outcomes

Subgroup analyses suggested that both the training dose and intervention duration may influence the therapeutic efficacy of BRT. Greater motor improvements were observed when the total training duration exceeded 15 h (SMD 0.27; 95% CI 0.11–0.43) and the intervention duration surpassed four weeks (SMD 0.37; 95% CI 0.05–0.69), whereas lower-dose programs did not yield significant benefits over control conditions. These findings suggest that sufficient training intensity and duration are required for BRT to produce measurable effects [54,55,56].

These improvements may be attributed to experience-dependent neuroplasticity and intensive motor learning, which require sustained, repetitive, and task-specific practice to induce neural reorganization [57]. Accordingly, BRT enables the structured delivery of high-intensity, high-repetition movement training [29,33,34], which may help explain the dose-dependent effects observed in the present meta-analysis. However, these findings should be interpreted cautiously, as heterogeneity in intervention protocols, comparator types, and participant characteristics across studies may have contributed to the observed variability. Overall, sufficient cumulative training appears essential for optimizing BRT outcomes.

### 4.6. Functional Outcomes and Clinical Implications

Although BRT demonstrated improvements in motor impairment under specific conditions, no significant advantages over control interventions were observed for activities of daily living, as measured by the Functional Independence Measure and Motor Activity Log. This dissociation between impairment-level and activity-level outcomes has been consistently reported in the stroke rehabilitation literature [58,59].

### 4.7. Limitations

Despite low statistical heterogeneity, notable clinical diversity was observed across studies in terms of robotic device designs and training protocols, which warrants cautious interpretation of the findings. Additionally, the limited number of trials evaluating specific robotic systems and the lack of functional and activity-level outcome data restrict conclusions regarding the comparative effectiveness of BRT. Finally, this meta-analysis pooled URT and CR into a single control group. Although these interventions differ conceptually, subgroup analyses did not demonstrate significant differences in treatment effects between comparator types. Nevertheless, this variation in control conditions should be considered when interpreting the comparative effectiveness of BRT.

## 5. Conclusions

This meta-analysis revealed that BRT did not provide a statistically significant overall advantage over URT or CR for upper-limb motor recovery after stroke. However, subgroup analyses suggest that treatment effects may vary according to patient characteristics, training dose, duration of the intervention, and device type.

## Figures and Tables

**Table 1:** Characteristics of the included randomized controlled trials.

Author	Experimental Intervention (Bilateral Robotic Training)				Control				Outcomes
	Intervention Description	N	Mean Age (SD)	FMA Mean (SD)	Intervention Description	N	Mean Age (SD)	FMA Mean (SD)	
Lum 2002 [29]	MIME + tone/positioning	13	63.2 (3.6)	24.8 (4.5)	CR = NDT therapy + tone/positioning	CR (*n* = 14)	CR: 65.9 (2.4)	CR: 26.6 (4.7)	FMA-UE, FIM, Barthel Index, upper-limb strength and 3D reaching distance
Hesse 2005 [30]	Bi-Manu-Track + NDT; gait/ADL; tone & proximal control	22	65.4 (11.5)	7.9 (3.4)	CR = ES + inpatient NDT-based PT/OT; gait/ADL; tone & proximal control	CR (*n* = 22)	CR: 64.0 (11.6)	CR: 7.3 (3.3)	FMA-UE, MRC, MAS
Lum 2006 [31]	MIME + bilateral rhythmic circular movements	5	69.8 (4.0)	38.6 (4.3)	URT = Unilateral MIME robotic training CR = Conventional therapy based on NDT principles	URT (*n* = 9) CR (n = 6)	URT: 69.8 (4.0) CR: 59.9 (5.5)	URT: 31.6 (2.7) CR: 26 (3.25)	FMA-UE, Motor Status Score, FIM, Motor Power Examination, MAS
Burgar 2011 [17]	(a) High-dose MIME (b) Low-dose MIME	17 19	58.6 (2.3) 62.5 (2.0)	19.0 (3.7) 26.7 (5.0)	CR = neuromuscular reeducation, resistive exercises, ADL	CR (*n* = 18)	CR: 68.1 (3.3)	CR: 24.2 (4.8)	FMA-UE, MRC strength, FIM, MAS, Wolf Motor Function Test
Hsieh 2011 [32]	(a) High-dose Bi-Manu-Track (b) Low-dose Bi-Manu-Track	6 6	56.0 (13.7) 52.4 (1.9)	44.0 (8.2) 37.7 (10.0)	CR = Structured conventional OT (NDT-based, strengthening, gross/fine motor, ADL training)	CR (*n* = 6)	CR: 54.0 (8.1)	CR: 37.5 (11.6)	FMA-UE, MRC, MAL, ABILHAND questionnaire, urinary 8-OHdG, MFSI
Liao 2012 [33]	Bi-Manu-Track	10	55.5 (11.2)	44.9 (9.0)	CR = Neurodevelopmental techniques; passive ROM; stretching; muscle strengthening; dexterity training; gross motor training	CR (*n* = 10)	CR: 54.6 (8.2)	CR: 39.6 (11.3)	Arm Activity Ratio, FMA-UE, FIM, MAL, ABILHAND questionnaire
Wu 2012 [34]	Bi-Manu-Track + functional tasks + tone normalization.	14	55.1 (12.7)	43.3 (10.1)	CR = weight bearing, stretching, strengthening, coordination, fine motor tasks, balance, and compensatory functional training	CR (*n* = 14)	CR: 51.3 (6.2)	CR: 45.4 (11.4)	Kinematic measurement, FMA-UE, MAL, SIS
Yang 2012 [35]	Bi-Manu-Track	7	51.4 (10.9)	41.9 (9.4)	URT = Bi-Manu-Track: paretic-arm only CR = Conventional upper-limb functional therapy	URT (*n* = 7) CR (*n* = 7)	URT: 50.8 (6.1) CR: 51.6 (7.6)	URT: 40.9 (6.4) CR: 43.3 (12.6)	FMA-UE, MRC, Grip Strength, MAS
Hsieh 2016 [36]	Bi-Manu-Track + Task-oriented training + functional tasks	16	49.3 (10.1)	26.8 (12.1)	CR = Phase 1 simple, high-repetition tasks, Phase 2 complex functional tasks	CR (*n* = 15)	CR: 52.9 (10.4)	CR: 29.1 (16.1)	FMA-UE, Grip Strength, Box & Block Test, Modified Rankin Scale, FIM, Wrist Actigraphy, SIS, Fatigue Scale
Hsu 2019 [37]	Bi-Manu-Track	22	53.1 (13.9)	38.6 (12.4)	CR = Sensorimotor stimulation + therapist-led task-specific training	CR (*n* = 21)	CR: 52.6 (12.5)	CR: 41.9 (14.9)	MAL, sEMG, FMA-UE
Hung 2019 [38]	Bi-Manu-Track + Bilateral task training + Home practice	10	62.6 (8.5)	31.9 (6.0)	URT = Bi-Manu-Track (unilateral mode) + unilateral arm training	URT (*n* = 9)	URT: 49.9 (10.6)	URT: 28.1 (5.5)	FMA-UE, CAHAI, GAS, MAS, VAS
Hung 2019 [15]	Bi-Manu-Track + bilateral arm training	15	58.4 (13.1)	31.6 (7.6)	URT = Bi-Manu-Track (unilateral mode) + unilateral arm training	URT (*n* = 14)	URT: 53.2 (12.3)	URT: 29.4 (7.1)	FMA-UE, SIS, ADL/IADL, WMFT, NEADL
Yuan 2023 [20]	Robot: ESTUN 3D upper-limb robot + functional tasks + ADL training	24	59.0 (8.3)	19.1 (9.2)	URT = ESTUN 3D upper-limb unilateral robot training (passive/assisted) + functional tasks + ADL training CR = FES, Bobath, hemiplegic limb training, ROM exercises, functional tasks, and ADL training	URT (*n* = 23) CR (*n* = 23)	URT: 56.7 (8.9) CR: 58.9 (10.3)	URT: 17.9 (11.6) CR: 18.6 (11.5)	FMA-UE, MBI, ADL, sEMG
Mauro 2024 [21]	ALEx RS exoskeleton, bilateral training	9	70.2 (4.9)	36.3 (13.9)	URT = ALEx RS exoskeleton, unilateral training	URT (*n* = 10)	URT: 68.9 (14.7)	URT: 35.2 (22.6)	HD-EEG, FMA-UE, ARAT, Motricity Index, MAS, WMFT, System Usability Scale, patient satisfaction

Abbreviations: ADL: activities of daily living; ARAT: action research arm test; CAHAI: Chedoke arm and hand activity inventory; CR: conventional rehabilitation; FIM: functional independence measure; FMA-UE: Fugl–Meyer assessment for upper extremity; GAS: goal attainment scaling; HD-EEG: high-density electroencephalography; MAL: motor activity log; MAS: modified ashworth scale; MBI: modified barthel index; MFSI: multidimensional fatigue symptom inventory; MIME: mirror image movement enabler; MRC: medical research council scale; NDT: neurodevelopmental treatment; NEADL: Nottingham extended activities of daily living scale; ROM: range of motion; SD: standard deviation; sEMG: surface electromyography; SIS: stroke impact scale; URT: unilateral robotic training; VAS: visual analog scale; 8-OHdG: 8-hydroxy-2′-deoxyguanosine.

**Table 2:** Overview of bilateral robotic training devices.

Device	Robot Type	Movement Capability	Primary Movements	Training Configuration	Clinical Focus
MIME	End-effector	6-DOF, three-dimensional workspace	Multijoint 3D reaching	Unilateral + bimanual mirror	Proximal control (shoulder–elbow)
Bi-Manu-Track	End-effector	2 × 1 DOF, distal cyclic movements	Forearm pronation–supination; wrist flexion/extension	Bilateral mirror cycles	Wrist–forearm repetition
ESTUN	End-effector	3D end-effector-driven	Shoulder & elbow movements	Unilateral or bilateral	Proximal reaching with support (shoulder–elbow)
ALEx RS	Exoskeleton	6-DOF exoskeleton	Shoulder + elbow 3D tasks	Unilateral or bilateral	Multijoint coordination + exergames (shoulder–elbow)

Abbreviations: 3D: three-dimensional; DOF: degrees of freedom.

**Table 3:** GRADE summary of findings: Bilateral Robotic Training versus Control.

Outcomes	No. of Participants (Studies)	Relative Effect (95% CI)	Certainty of the Evidence (GRADE)	Anticipated Absolute Effects/Comments
Upper-limb motor function (FMA-UE)	440 (14 RCTs; 16 comparisons)	SMD 0.18 (−0.01–0.36)	●●○○ LOW ^ab^	The results suggest a small positive effect of bilateral robotic training on upper-limb motor function; however, the certainty of evidence is low, and the confidence interval includes no effect.

^a^ Risk of bias: The risk of bias was downgraded by one level due to a serious risk of bias in several included studies, which was related primarily to deviations from intended interventions and selective outcome reporting. ^b^ Imprecision: Downgraded by one level because the 95% confidence interval includes no effect and the pooled effect estimate is small. ●●○○ indicates low certainty of evidence.

## Data Availability

The data analyzed in this study were extracted from previously published articles included in the systematic review.

## Supplementary Materials

The following supporting information can be downloaded at: https://www.mdpi.com/article/10.3390/medsci14020293/s1, Supplementary S1: Search strategy; Supplementary S2 Table S1: Summary of adverse events and attrition rates in the included studies.
