# Supplementary material for: Effects of Bilateral Robotic Arm Training in Stroke Patients: A Systematic Review and Meta-Analysis
Source: Med Sci (Basel). 2026 Jun 5;14(2):293. doi: 10.3390/medsci14020293 (PMC13302995; doi:10.3390/medsci14020293)
Supplement: Supplementary file 1 [file medsci-14-00293-s001.zip › medsci-4235782-supplementary/Supplementary 1 Search Strategy .docx]

Supplementary 1.

Search Strategy

*Search Strategy for PubMed*

1. Search terms for Condition (Population): (“Stroke” [Text word] OR “Hemiparesis” [Text word] OR “Upper extremity impairment” [Text word] OR “Upper extremity paresis” [Text word] OR “Upper limb motor Deficits” [Text word] OR “Cerebrovascular accident” [Text word] OR “Subacute stroke” [Text word]) OR (“Stroke” [MeSH Terms] OR “Upper extremity” [MeSH Terms] OR “Paresis” [MeSH Terms])

2. Search terms for intervention: ("Robot-assisted upper-limb therapy" [Text word] OR "Robot rehabilitation" [Text word] OR "Bilateral robotic" [Text word] OR "Bilateral priming" [Text word] OR "Bilateral hybrid" [Text word] OR "Hybrid rehabilitation" [Text word] OR "Robot-assisted upper limb" [Text word] OR "Robot-assisted movement training" [Text word] OR "Robotics" [Text word] OR "Robotic device" [Text word] OR "Robot-assisted bilateral arm training" [Text word] OR “Stroke rehabilitation” [Text word] OR “Bilateral robotic priming” [Text word]) OR ("Robotics" [MeSH Terms])

3. Search terms for Outcome: (“Fugl–Meyer Assessment” [Text word] OR “Upper extremity motor function” [Text word] OR “Upper-Limb Function” [Text word] OR “Fugl–Meyer Assessment Scale” [Text word] OR “Upper-extremity performance” [Text word]) OR (“Motor activity” [MeSH Terms] OR “Recovery of function” [MeSH Terms])

**The final search strategy was as follows:**

Combine 1, 2, and 3, and use the Boolean operator AND to retrieve articles relevant to the condition, intervention, and outcome

**Search Strategy for the Cochrane Library**

The following MeSH terms were used to search the Cochrane Library:

#1 MeSH descriptor: [Stroke] explode all trees

#2 MeSH descriptor: [Upper Extremity] explode all trees

#3 MeSH descriptor: [Robotics] explode all trees

Final search combination: #1 AND #2 AND #3

**Search Strategy for Google Scholar**

Searches via keywords:

stroke AND “bilateral robot* “ AND “upper extremity”

**Search Strategy for Physiotherapy Evidence Database (PEDro)**

Search terms used:

robot* AND stroke

Search Strategy for Scopus

Search string:

robot* AND stroke AND “upper limb”

Search Strategy for Embase

(stroke OR “cerebrovascular accident” OR hemiplegia OR poststroke) AND (robot* OR “robotic rehabilitation” OR “robot-assisted” OR exoskeleton) AND (bilateral OR “both arms” OR “two arms” OR “dual arm” OR “upper limbs” OR “upper extremity”) AND (training OR rehabilitation OR therapy OR exercise)

The asterisk (*) enables the retrieval of all related terms, such as *robotic*, *robot-assisted*, etc.
