# Supplementary material for: Effects of Bilateral Robotic Arm Training in Stroke Patients: A Systematic Review and Meta-Analysis
Source: Med Sci (Basel). 2026 Jun 5;14(2):293. doi: 10.3390/medsci14020293 (PMC13302995; doi:10.3390/medsci14020293)
Supplement: Supplementary file 1 [file medsci-14-00293-s001.zip › medsci-4235782-supplementary/Supplementary 2 Adverse event.docx]

**Supplementary 2.**

| **Study** | **Adverse events** | **Post-intervention drop-out (n, %)** | **Reasons for drop-out** |
| --- | --- | --- | --- |
| Lum 2002 | Not reported | 3/30 (10.0%) | Medical complications unrelated to study (n=2); not stroke diagnosis (n=1) |
| Hesse 2005 | No serious adverse events reported; mild hand swelling observed in ES group (n=3) | 5/44 (11.4%) | Refused therapy (n=1); loss to follow-up (n=4) |
| Lum 2006 | Not reported | 1/30 (3.3%) | Reasons unrelated to the study |
| Burgar 2011 | Not reported | Not reported | None |
| Hsieh 2011 | No serious adverse events reported; fatigue levels were low | 0/18 (0%) | None |
| Liao 2012 | No adverse events reported | 0/20 (0%) | None |
| Yang 2012 | Not reported | 0/21 (0%) | None |
| Wu 2012 | Not reported | 0/42 (0%) | None |
| Hsieh 2016 | No serious adverse events; mild fatigue both group, well tolerated | 0/31 (0%) | None |
| Hsu 2019 | No adverse events reported | 0/43 (0%) | None |
| Hung 2019 | 1 mild low back pain (BHT, resolved); 1 device-related hand pain (RT, acceptable); fatigue acceptable; no serious adverse events | 0/30 (0%) | None |
| Hung 2019 | No adverse events reported | 1/45 (2.2%) | Medical reasons (unrelated to intervention) |
| Yuan 2023 | Not reported | 9/79 (11.4%) | Refusal to undergo evaluation |
|  |  |  |  |
| Mauro 2024 | Not reported | 1/19 (5.3%) | Did not complete protocol (<30 sessions), unrelated reason |

**Table S1.** Summary of adverse events and attrition rates in the included studies.

Note. BHT = bilateral hybrid therapy; RT = robot-assisted therapy.
